# Psychosocial interventions for adults with newly diagnosed chronic disease: A systematic review

**DOI:** 10.1177/1359105321995916

**Published:** 2021-02-14

**Authors:** Samuel Akyirem, Angus Forbes, Julie Lindberg Wad, Mette Due-Christensen

**Affiliations:** 1 King’s College London, UK; 2 SDA Nursing and Midwifery Training College, Asanta, Ghana; 3 Steno Diabetes Center Copenhagen, Denmark

**Keywords:** adaptation, chronic disease, coping, newly diagnosed, psychosocial intervention, social support

## Abstract

While the need for psychosocial interventions in the early formative period of chronic disease diagnosis is widely acknowledged, little is known about the currently available interventions and what they entail. This review sought to collate existing interventions to synthesize their active ingredients. A systematic search on five electronic databases yielded 2910 records, 12 of which were eligible for this review. Evidence synthesis revealed three broad categories of interventions which used at least two out of eight active techniques. Future studies should adhere to known frameworks for intervention development, and focus on developing core outcome measures to enhance evidence synthesis

## Introduction

The prevalence of chronic diseases is increasing (Helgeson and Zajdel, 2017). Chronic diseases can have a significant impact on a person’s life habits and relationships, as they often require lifestyle changes and the adoption of self-management behaviours required to reduce the physical effect of the disease. Hence, being diagnosed with a chronic disease can be life-changing and precipitate multiple physical, psychological and social sequelae. Therefore, the support provided to people at the point of diagnosis is important. This support not only needs to ensure that the person can manage the physical impact of the disease, but also the psychological and social challenges it brings.

A new diagnosis of chronic disease has been likened to a crisis situation that produces initial responses of fear, anxiety, depression and anger (Martz and Livneh, 2007). The individual may experience grief due to the permanent loss of health (Burke et al., 1992). A new diagnosis may also present with shock, re-evaluation of one’s future, stigma and negative effects on relationships (Due-Christensen et al., 2018). The emerging physical symptoms and associated self-management requirements of a new-onset chronic disease may provoke significant changes in routines and established lifestyle habits often in a sudden manner allowing limited time for adjustment.

Moreover, the uncertainty surrounding the cause and course of some chronic diseases may also be unsettling for patients as individuals may appraise the unknown as an outcome that engenders fear and anxiety (Carleton, 2016).

Socially, chronic diseases may expose individuals to stigma and discrimination (Browne et al., 2014; Earnshaw et al., 2012; Lempp et al., 2006). The disease may limit an individual’s ability to engage in interpersonal relationships perhaps due to fear of being misunderstood (Gignac et al., 2013). In addition, the physical limitations imposed by chronic diseases may limit individuals’ ability to contribute their quota in reciprocal social relations; straining their relationships and potentially causing feelings of guilt in patients for being a burden on their family or friends (Trindade et al., 2018; Williams and Wood, 1988). These social issues may be distressing for patients.

Adaptation to chronic disease is therefore essential as the lack thereof may result in several psychological and social challenges for patients. Adaptation is a complex and dynamic process (Helgeson and Zajdel, 2017; Stanton et al., 2007) that requires cognitive, affective and behavioural efforts (Dekker and de Groot, 2018). The process involves coming to terms with the disease and modifying one’s lifestyle to suit or conform to the new situation (De Ridder et al., 2008). Several theories underpin the process of adaptation. The primary theories highlight the importance of illness perception (Leventhal et al., 2016), benefit finding (Moss-Morris, 2013), self-efficacy and stress reappraisal (Lazarus and Folkman, 1984) in psychosocial adaptation. Psychosocial interventions target these theoretical elements to enable patients to achieve adaptation.

The process of adaptation may be daunting for patients. Therefore, a support system (in the form of psychosocial intervention) may be required to equip patients with the skills to navigate life with chronic disease (Purgato et al., 2016). The first few years of diagnosis are considered formative and critical in determining patients’ perceptions of their disease and the success of adaptation. Interventions that target this formative period may have a higher chance of preventing poor physical and psychosocial outcomes (Martz and Livneh, 2007). While the need for psychosocial interventions in the early formative period of chronic disease diagnosis is widely acknowledged, little is known about the currently available interventions and what they entail.

Psychosocial interventions are likely to have several components and modes of delivery and would often be complex (Deary et al., 2018). For such interventions, precise specifications of intervention facets are required to build the evidence base that will inform subsequent development and delivery of effective interventions (Craig et al., 2008).

The current review sought to collate existing psychosocial interventions for adults with newly diagnosed chronic disease and to synthesize their active ingredients to provide the evidence base for the modelling of future effective interventions for this population.

## Methods

The current review was conducted in line with Joanna Briggs Institute’s (JBI) guidelines for systematic reviews (Tufanaru et al., 2017). JBI guidelines involve formulating a review question, defining inclusion and exclusion criteria, locating studies through searching, selecting studies for inclusion, assessing the quality of studies, extracting data, synthesizing the relevant studies and presenting and interpreting the results. The review was registered on PROSPERO (CRD42020163806).

### Review questions

The review sought to answer four questions:

- What are the various psychosocial interventions for patients with newly diagnosed chronic disease?
- What theoretical models underpin these interventions?
- What are the active ingredients of the interventions?
- How *efficacious* are the interventions at improving physical, psychological, social and affective outcomes?

### Defining inclusion and exclusion criteria

The following eligibility criteria were used: (a) use of psychosocial intervention intended to facilitate psychosocial adaptation (b) studies of all designs (c) adult participants (>18 years) with multiple sclerosis (MS), rheumatoid arthritis (RA), type 1 diabetes (T1D), type 2 diabetes (T2D), HIV or inflammatory bowel disease (IBD) (d) disease duration of 3 years or less (e) reporting at least one psychosocial outcome (f) papers reported in English. We focused on only six chronic diseases for pragmatic reasons. These chronic diseases were selected because of the extensive self-management practices they require in the form of adhering to complex drug regimens, dietary restrictions and adjusting to the psychosocial and physiological demands of the disease, that are likely to put undue psychological stress on patients (Grady and Gough, 2014). This review excluded evidence from the grey literature.

### Locating studies through searching

An electronic search was conducted on OVID EMBASE (1974–2020 Week 21), MEDLINE (1946 to 20th May 2020), PsychInfo (1806 to May Week 4 2020), PUBMED and EBSCO CINAHL from inception to date in line with the JBI guidelines for literature search (Aromataris and Riitano, 2014). Facet analysis and search terms are depicted in Table 1. Boolean combinations of search terms were applied (‘OR’ for terms in the same column, ‘AND’ for terms across columns). The search strategy was modified for each electronic database details of which are presented in Supplemental S1.

**Table 1.** Facet analysis of review question.

	Population 1	Population 2	Intervention
Index term (exploded)	HIV/AIDS	–	Psychosocial support
	Rheumatoid arthritis		Psychotherapy
	Multiple sclerosis		Psychoeducation
	Type 1 diabetes mellitus		Patient support
	Type 2 diabetes mellitus		Acceptance and commitment therapy
	Inflammatory bowel disease		Cognitive behavioural therapy
Free text, synonyms, alternative spelling and abbreviations	HIV infection	Recent diagnosis	CBT
	Retroviral infection	New onset	ACT
	Rheumatism	New diagnosis	Patient education
	Crohn’s disease	Recently diagnosed	Acceptance
	Ulcerative colitis	Newly diagnosed	Commitment
	T1D, T2D, PLWH, PLWHA, IBD	After diagnosis	

In addition, references of retrieved studies were hand-searched to identify other relevant studies not captured by the electronic search.

### Selecting studies for inclusion

Screening and selection of relevant studies were guided by the JBI guidelines for study selection (Porritt et al., 2014). The results of the electronic search were imported into Covidence (https://www.covidence.org) to facilitate the screening process. Duplicates were automatically removed. One reviewer (SA) performed title and abstract screening against the eligibility criteria. A sample of one hundred (100) studies was taken by a second reviewer (MD) to validate the screening process. A third reviewer (AF) was contacted where there were disagreements. Full-text review of tentatively eligible studies was then carried out to identify studies that met the inclusion criteria.

### Assessing the quality of studies

JBI Critical Appraisal Checklist for Quasi-Experimental (JBI-QE) and randomized controlled trials (JBI-RCT) studies were used for non-RCTs studies and RCTs respectively (Tufanaru et al., 2017).

The overall quality of RCTs was adjudged ‘high’ (study reports 10 or more items on the checklist including randomization, blinding, concealment and power calculations), ‘moderate’ (study reports seven or more items on the checklist including at least one of the following: randomization, blinding or concealment) or ‘low’ (study reports fewer than six items). For quasi-experiments, it was determined that the highest possible overall quality of quasi-experiments will be ‘moderate’ (reports seven or more items on the checklist including the use of a comparison group). Quasi-experiments with no control groups were adjudged to have ‘low’ quality even if all other items on the checklist are reported.

Studies were not excluded based on methodological quality because of the focus (primary) of this review on intervention components and delivery rather than *efficacy.*

### Extracting data

Data extracted included: authors, year of publication, country, sample characteristics, intervention components, mechanism/theory of action, main outcomes and limitations. Intervention details were extracted with the COMPASS (COMPlex interventions: Assessment, trialS and implementation of Services) checklist for psychological interventions (Hodges et al., 2011). COMPASS checklist helps to define complex interventions by specifying the context, content, mechanism of action, target outcomes and method of delivery.

### Synthesizing the relevant studies

Tabular synthesis of key features (e.g. study designs, sample characteristics, type of interventions, mechanism of action, findings) of included studies was undertaken.

In addition, theoretical synthesis (Forbes and Griffiths, 2002) was used to identify the various components of psychosocial interventions. Firstly, intervention contents (including techniques, timing, therapists, etc.) as extracted with the COMPASS checklist, were loaded into NVIVO 12 (QSR International, Melbourne, Australia). Deductive and inductive thematic analysis of intervention contents were then performed recursively to identify commonalities in the active techniques used by the various studies. The deductive analysis was informed by the literature on adaptation to chronic diseases. For instance, initial coding was centred on the concepts of acceptance and stress reappraisal which are important to psychosocial adaptation. Themes were then refined and defined. A similar approach was used to identify the type, setting and timing of interventions.

The clinical heterogeneity of the included studies precluded the pooling of outcome measures for meta-analysis. A narrative approach to synthesis, utilizing textual descriptions to summarize key study outcomes (Tufanaru et al., 2017) was undertaken to summarize the impact of the various psychosocial interventions on physical, affective, psychological and social outcomes.

## Data sharing statement

As a systematic review, the datasets we used were already published articles which are accessible online. References to such articles are provided as appropriate throughout this paper. To ensure replicability of this review, specific search terms used on each of the five databases and the corresponding number of results are provided in Supplemental S1. Detailed data extraction forms for each included study are also provided. Moreover, the NVIVO file which was generated during the synthesis of intervention facets is also attached. These files are accessible via FigShare repository.

## Findings

### Results of the search

**Figure 1.**
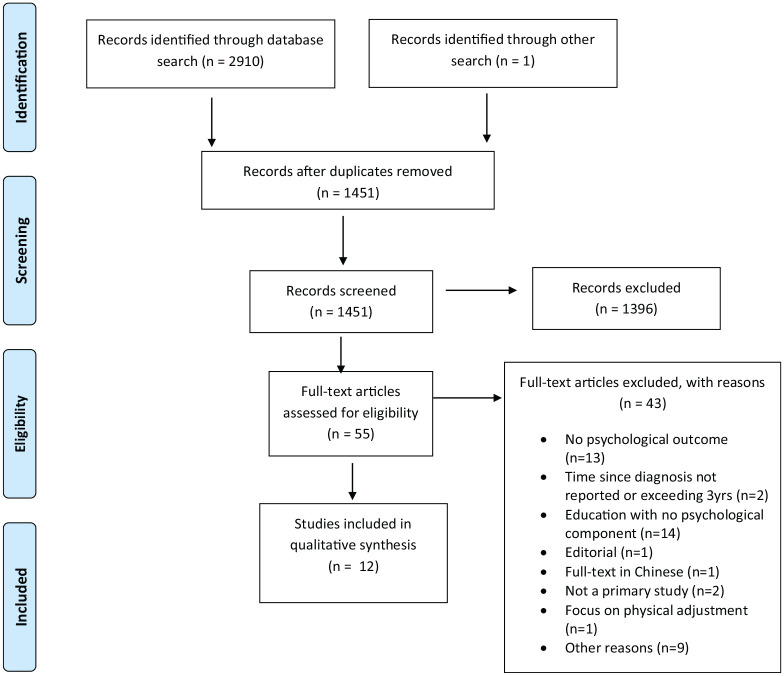
PRISMA chart (Moher et al., 2009).

The electronic searches yielded 2910 records. One additional record was identified through reference searching. After removal of duplicates, 1451 papers remained. A total of 1396 papers were excluded after the title and abstract screening. The majority of those studies were educational and self-management interventions. Full texts of the remaining 55 papers were reviewed. Fourty-three (43) records were excluded for reasons stated in the Preferred Reporting Items for Systematic reviews and Meta-Analysis (PRISMA) chart (Figure 1). Reasons for the exclusion of nine additional papers (as shown in the PRISMA chart) are presented in Table 2. Twelve (12) quantitative papers that met the eligibility criteria were included in this review.

**Table 2.** Studies excluded for other reasons.

Studies	Reasons for exclusion
Feicke et al. (2014)	The intervention described in this study appeared to have a psychosocial component. The focus, however, was on increasing self-management skills.
Moskowitz et al. (2012)	A pilot study. The main study which was conducted in 2017 was included in this review instead.
Govender et al. (2014)	This study was about a suicide prevention intervention that seemed too specific and peculiar to HIV. It was excluded based on its relevance to the overarching objective of this review.
Gafvels et al. (2014); Rane and Gafvels (2017)	The studies evaluated intervention by a medical social worker. Different types of tailored psychosocial interventions were used but studies provided no details of such interventions. Therefore, they could not contribute to the overarching objective of this review.
Sharpe et al. (2001, 2003)	These two studies represent a case series (2001) and the 2-year follow-up (2003) reports of a 2001 study (included in this review). The follow-up report provided data on the long-term impact of the psychosocial intervention. However, the lack of intention-to-treat analysis and inconsistencies with the main 2001 study led to its exclusion from this review.
Harper et al. (2014)	Non-adult participants (<18 years) were included in this study
Whittemore et al. (2001)	This study describes a nurse coaching intervention aimed at increasing adherence to dietary and lifestyle modification following an educational programme. Excluded for its focus on lifestyle changes and no clear psychological component.

### General characteristics of included studies

Table 3 summarizes the key features and findings of the included studies. The 12 included studies had a combined sample size of 900 (range 9–279) and a median follow-up period of 7 months (range 1–15 months). A majority of the studies were of RCT design with five (5) being quasi-experimental studies and one with unspecified study design (Brusadelli et al., 2018). Four studies originated from the USA, two from Italy and one each from the UK, Australia, Austria, The Netherlands, China and South Africa. Majority of the studies were on HIV (*n* = 5), followed by MS (*n* = 4), with RA, T1D and T2D contributing one study each. No study was found for IBD. The average duration since diagnosis ranged from 4.3 weeks to 3 years. Studies were published between 1995 and 2019 with most published within the past 10 years (*n* = 9).

**Table 3.** Summary of evidence.

First author, (year), country, design	Objective	Sample characteristics	Intervention/underlying theory	Active ingredients or mechanism of action	Major findings	Additional comments	Quality assessment
Spiess (1995), Austria, RCT	To determine the impact of a distress reduction intervention on psychosocial variables and the association between adaptation and HbA1c	T1D with DD (m, SD) = 4.3 (4.2) weeks; IG: *n* = 10 (four females); age = 24.6 (5.29); HbA1c – 10.1 (1.6) %; CG: *n* = 13 (five females); age = 24.46 (4.11); HbA1c – 10.5 (1.8) %	Onset distress reduction programme – group format, psychotherapist-led and having a total of 25 90 minutes-sessions + self-management education*N/R*	Emotional and grief expressionAddressing anxieties regarding late complications.Challenges of living with diabetes as well as the impact on social and family life and coping strategiesCognitive restructuring	HbA1c improved in IG/CG groups (6.9%/6.6%) at 15 months follow up with no significant difference between groups.Depression, anxious coping and denial score improved at 9 months in the intervention group only (p<0.01).	90% attended between 13 and 23 groups sessions. Participants in group sessions not stratified by, for instance, age group	Moderate

					At 15 months, the effects of all psychosocial measures in the intervention group had faded sparing denial.		
Mundell (2011), South Africa, Quasi-experimental	To assess the impact of a psychosocial support group on pregnant HIV positive women	HIV with DD (m, SD): 13.97 (46.02) weeks; IG: *n* = 129; age = 27.35 (5.49); CG: *n* = 150; age = 26.89 (5.34); partnered *n* = 136	Support group – psychology students-led, and having a total of 10 sessions	Education on HIV and its emotional impact. Addressing relational issues such as disclosure, stigma and discrimination. Coping strategies and stress management were addressed. Life planning and goal setting	Accelerated increase in coping (MD-2.68, *p* = 0.01) and self-esteem (MD-1.92, *p* = 0.06) from baseline to the first follow-up in IG. At the final follow-up, the associated scores of the comparison/intervention group had reached similar levels.	Improvement was based on attendance as those who attended five or more sessions had better outcomes. Therapists were trained and intervention fidelity ensured	Moderate
			*Hanson’s System theory framework*				
Moitra (2015), Quasi-experiment (no control), USA	To determine the feasibility and acceptability of an acceptance-based intervention and its impact on experiential avoidance.	HIV with DD (m, SD) = 84.7 (76.3) days; *n* = 9 (three females); age = 33.1(11.9)	Acceptance-Based Behaviour therapy (ABBT) – individual format, clinical psychologist-led, having a total of two sessions and lasting for 15–20 minutes each	Psychoeducation. Cognitive defusion and experiential acceptance techniques. Focus on life goals to provide a sense of coherence for patients.	HIV acceptance increased from baseline to 1-month follow-up (MD on the AAQ scale– 4.21). Also, reductions in depressive symptoms (MD on PHQ scale – 0.38), HIV health care system distrust and stigmatization (MD on HSS scale – 1) were detected post-intervention.	Three participants refused the intervention because they felt too emotionally distressed about their new diagnosis. [how long after diagnosis is it appropriate to start intervention?]	Low
			*Hayes’ Relational Frame theory*				
Sharpe (2001), UK, RCT	To investigate whether a CBT applied within the first 2 years of illness could be effective in reducing psychological morbidity in RA patients	RA with DD (m, SD) – 12.6 (14.1) months; IG: *n* = 27 (16 females); age = 54.13 years; CG: *n* = 26 (16 females); age = 56.86 years	CBT – individual format, psychologist-led and having a total of eight 1 hour sessions + Routine medical care	An educational component on the management of flare-ups or high-risk situations, Self-management skills, Relaxation training, attention diversion, goal setting, pacing, problem-solving, cognitive restructuring and enhancing communication skills.	Clinically significant improvements in HADS scores in IG (17% to 4% decrease in ‘possible cases of depression’ in this group). Non-significant changes in anxiety were observed (*p* = 0.068). There was a transient reduction in C-reactive protein, although the difference between the CBT group and the others was not maintained after 6 months	Treatment manual used. Dropouts were younger, more depressed and had a higher level of joint dysfunction than those completing treatment.	Moderate
			*Beck’s cognitive theory*				
Yang (2018), China, Quasi-experiment (no control)	To assess pre-post mental health outcomes of CBT for gay men recently diagnosed HIV patients	HIV with DD (median): 66 days; *n* = 10 (all males); age = 26.5 (8.0) years	CBT – individual format, psychologist-led and having a total of three sessions of unspecified lengths.*Beck’s cognitive theory*	Relaxation techniques, cognitive restructuring, problem-solving and keeping automatic thought records to keep track of negative thoughts.	Significant improvements in depression (Cohen’s *d* = 0.82, *p* = 0.03), HIV coping (Cohen’s *d* = 1.63, *p* = 0.001) and total social support (Cohen’s *d* = 1.05, *p* = 0.009) were observed.	Text messages used to remind participants of sessions. Intervention fidelity was ensured.	Low

Brashers (2017), USA, RCT	To assess the efficacy of peer support intervention designed to improve uncertainty management and psychosocial functioning for patients newly diagnosed with HIV	HIV with DD (IG/CG): 1.5/1.8 years; IG: *n* = 54 (four females); age = 36 years; CG: *n* = 44 (10 females); age = 36.55 years	Uncertainty management intervention – peer educator-led, unclear format, with a total of six 1 hour sessions*Brasher’s Uncertainty Management theory*	Psychoeducation. Where and how to find pertinent information. Effective communication with family, friends and healthcare workers	A decline in illness uncertainty in the IG compared to the CG was observed at all follow-up periods. No improvement was seen in the perceptions of available social support. Improvement in depression scores was seen in IG whereas those in the CG experienced no significant change in depression over time. Self-advocacy did not change in either group.	Peer educators were trained before the intervention. Participant reimbursements were used to attract information avoiders to participate	Low
Calandri (2017), Italy, Quasi-experimental	To evaluate a group-based cognitive behavioural intervention to promote the quality of life and psychological well-being of patients with newly diagnosed MS.	MS with DD (IG/CG) – 1.5/1.8 years, IG: *n* = 54 (33 females), age – 38; CG: *n* = 31 (17 females), age = 34.8	CBT – group format, psychologist-led and having five 2 hour sessions with additional sessions at 6- and 12-months follow-up periods	Relaxation exercises. Exploring identity change and redefinition of life goals. Goal setting, managing symptoms and illness-related negative emotions. Cognitive restructuring, effective, communication and homework assignment.	At 6 months, the mental health component of QoL increased in IG (*p* = 0.036) but reduced in CG. Negative affect decreased in IG and increased in CG (*p* = 0.048). Optimism increased in IG and CG. Statistically non-significant Improvements were seen in physical health, positive affect and depression scores. All observed effects were maintained at 12-month follow-up except for optimism which declined in CG.	The intervention group was stratified by age-groups to facilitate the sharing of similar experiences. Each session had a 15 minutes break	Moderate
			*Beck’s cognitive theory*				
Visschedijk (2004), Netherland, Quasi-experimental	To estimate the effect of a cognitive-behavioural based group intervention programme on health-related quality of life in patients with MS	MS with DD – <3 years, *n* = 11 (eight females), Age = 38 years	CBT – group format, psychologist-led and having a total of eight 2 hour sessions.	Moving on after diagnosis, self-management, efficient communication with family, friends and medical staff. Coping with psychological distress, uncertainty and fear	Improvement in psychological status (ES – 0.29, *p* = 0.01) and vitality (ES – 0.49, *p* = 0.04) immediately post-intervention. No significant changes were seen at 6- and 12-months follow-up.	Two patients dropped out because confrontation with other patients in a group setting was too distressing for them.	Low
			*Beck’s cognitive theory*				
Moskowitz (2017), USA, RCT	To determine the impact of positive affect intervention on positive emotion, psychological health, physical health and health behaviours in people newly diagnosed with HIV.	HIV with DD – 2 months, IG: *n* = 80 (seven females), age = 35.6, CG: *n* = 79 (four females), age = 36.5	Positive affect intervention – individual format, led by facilitators with experience in public health research. A total of five 1 hour sessions in addition to a follow-up phone call on week 6.*Fredrickson’s Broaden-and-build theory of positive emotions*	Positive reappraisal, benefit finding and capitalizing on the positives of the disease. Eliciting positive feelings by showing gratitude and being kind to others despite physical limitations. Relaxation techniques such as mindfulness.	No significant change in negative affect and CD4 in IG/CG at 15 months. Intrusive and avoidant thoughts improved more in IG than CG (ES – 0.29, *p* = 0.047) at 15 months	72% retention rate despite reimbursing participants for attending treatment and assessment sessions.	Moderate
						Therapists were extensively trained, and intervention fidelity was ensured.	
Kiropoulos (2016), Australia, RCT	To examine the effectiveness and acceptability of a tailored CBT intervention for the treatment of depressive symptoms in those newly diagnosed with MS.	MS with DD (IG/CG) – 26.2/23.53 months; IG: *n* = 15 (13 females), age = 34.6 years; CG: *n* = 15 (nine females), age = 39.27,	CBT – individual format, psychologist-led, with eight sessions lasting between 1 and 1.5 hours each	Progressive muscle relaxation, controlled breathing exercises, pleasant activity scheduling, problem-solving skills, cognitive exercises which helped individuals identify, challenge and manage unhelpful thoughts and beliefs. Homework for each session to reinforce learned skills	CBT group had significantly lower depression scores at post-treatment (ES = 1.66) and at 20 weeks follow up (ES = 1.34) compared to the CG. Anxiety was lower in the CBT group though non-significant. Physical (ES – 0.7) and mental health (ES – 1) components of QoL were significantly higher in the CBT group than TAU at 20 weeks	A therapy manual was used.	Moderate
			*Beck’s cognitive theory*				
Molton (2019), USA, RCT	To develop and test a brief intervention designed to improve the ability to tolerate uncertainty and to improve MS acceptance in individuals in the early phases of MS	MS with DD – 376.3 days; IG: *n* = 23 (16 females); age = 39.61; CG: *n* = 25; age = 35.92	Psychological Intervention led by ‘study clinician’, delivered in an individual format and comprising six sessions.	General education, Mindfulness/thought awareness to enable patients to deal with rumination and catastrophizing. Managing the controllable and accepting the uncontrollable aspects of MS. Empowering patients to focus on their life goals/values and to pursue them despite their chronic disease.	Postintervention, those in the intervention group demonstrated lower levels of IU (Cohen’s *d* = 0.6) and more MS acceptance (Cohen’ *d* = 0.8) relative to the TAU group. There was no effect of the intervention on global anxiety. Decreases in IU were associated with increases in MS acceptance (*r* = −.63). The effect sizes for these changes were moderate.	A treatment manual was used. Strategies to increase participation included use of a few sessions and the option to receive treatment either via face-to-face or telephone	Moderate
			*A blend of Relational frame theory and Cognitive theory*				
Brusadelli (2018), Italy, Unclear	To evaluate the effects of psychological intervention on HbA1c and psychosocial outcomes in newly diagnosed T2DM	T2D with DD <12 months; *N* = 95 (IG = 47, CG = 48)	CBT-like intervention, group format, psychologist-led, with six 90 minutes sessions	psychoeducation (including recommendations for a healthy lifestyle and diet), problem-solving, cognitive restructuring and enhancement of emotional communication.	Higher clinically significant improvement in HbA1c was seen in IG (*d* = 0.77) than the CG (*d* = 0.35) at 6 months. Improvements in HbA1c were maintained in the IG but not the CG at 12 months follow up. Psychosocial outcomes were not reported.	–	Low
			*N/R*				

IG: intervention group; CG: control group; m, SD: mean, standard deviation; N/R: Not reported; T1D: Type 1 diabetes; T2D: Type 2 diabetes; RA: Rheumatoid arthritis; MS: Multiple sclerosis; HbA1c: Glycated haemoglobin; DD: disease duration; ES: effect-size; CBT: cognitive behavioural therapy; MD: mean difference; ESR: Erythrocytes sedimentation rate; TAU: treatment as usual: HADS: Hospital anxiety depression scale; HSS: HIV Stigma Scale; PHQ: Patient health questionnaire; GAD-7: general anxiety scale; QoL: Quality of life; AAQ: Acceptance and Action Questionnaire; EDSS: Expanded Disability Status Scale.

### Quality assessment

The quality of the included studies ranged from low to moderate. For the quasi-experiments, baseline characteristics for studies with controls were similar in the treatment/comparison groups except for one study that differed at baseline on per capital income/employment/HIV disclosure (Mundell et al., 2011).

All six RCT studies reported the use of randomization but only two studies outlined how allocation sequences were generated. Baseline characteristics of some studies (Brashers et al., 2017; Molton et al., 2019; Sharpe et al., 2001) differed for the intervention and control groups, thereby increasing the risk of selection bias. Allocation concealment and blinding of outcome assessors were used by four studies to minimize performance bias. The nature of the interventions did not allow for the blinding of interventionists and participants and this could have resulted in an overestimation of effect sizes. Intention-to-treat (ITT) analysis was used by all RCTs except for one study (Brashers et al., 2017). Studies generally reported a low attrition rate. Only two studies discussed how they determined their sample size (Moskowitz et al., 2017; Sharpe et al., 2001). Few studies reported measures of effect size to indicate the magnitude of any improvements attributable to the intervention.

### Synthesis of evidence

#### Intervention types and their theoretical underpinnings

Three categories of psychosocial interventions were identified namely, CBT/CBT-like, uncertainty management and social support interventions. Five studies utilized CBT interventions based upon the assumptions that one’s thought influences their emotions and behaviours, that is, Beck’s cognitive theory (Beck, 2005).

CBT-like interventions were used by five studies and they included positive affect interventions (PAI), acceptance and commitment therapies (ACT), intolerance to uncertainty intervention (a blend of ACT and CBT) and a distress reduction programme with an unspecified theoretical framework (Spiess et al., 1995). PAI was based on Fredrickson’s broaden-and-build theory of positive emotions (Fredrickson, 1998) which posits that certain positive emotions such as contentment can produce enduring improvements in patients’ psychological resilience, coping resources, intellectual capacity as well as the reversal of lingering negative emotions (Fredrickson, 2001, 2004). ACT was based on Hayes’ Relational frame theory (Hayes et al., 2001). The goal of ACT was to help individuals change their relationship with their thoughts as opposed to changing the contents of such thoughts.

Uncertainty management intervention (UMI) based on Brasher’s uncertainty management theory (2001) was used by one study (Brashers et al., 2017). UMI differed from intolerance to uncertainty interventions which rather focused on promoting acceptance to the disease. The guiding principle of the uncertainty management intervention was that enhancing patients’ communication skills facilitate adaptation to chronic disease.

Social support intervention (SSI) was a peer-led programme that emphasized vicarious experiences (Mundell et al., 2011). Hanson’s systems theory framework underpinned the SSI (Hanson, 1995).

#### Active techniques used

Overall, eight distinct active techniques were identified through the thematic analysis of the intervention contents. As shown in Table 4, each study utilized an average of four of these techniques (range 2–6).

**Table 4.** Active ingredients of psychosocial intervention.

	Brashers et al.	Brusadelli et al.	Calandri et al.	Kiropoulos et al.	Moskowitz et al.	Moitra et al.	Moltron et al.	Mundel et al.	Sharpe et al.	Spiess et al.	Visschedijk et al.	Yang et al.
**Type of intervention**												
CBT or CBT-like		✓	✓	✓	✓	✓	✓		✓	✓	✓	✓
Uncertainty management	✓											
Social support								✓				
Active techniques												
Education	✓	✓	✓			✓	✓	✓	✓	✓	✓	
Communication development	✓	✓	✓					✓	✓	✓	✓	
Relaxation techniques			✓	✓	✓	✓		✓	✓			✓
Cognitive restructuring		✓	✓	✓	✓				✓		✓	✓
Homework			✓	✓	✓	✓					✓	✓
Problem-solving		✓		✓					✓	✓	✓	
Acceptance and finding meaning			✓			✓	✓	✓		✓	✓	
Goal setting					✓			✓	✓			
Timing of intervention												
Brief	✓	✓	✓		✓	✓	✓					✓
Long-term				✓				✓	✓	✓	✓	
Location												
Medical	✓			✓		✓	✓		✓		✓	✓
Non-medical			✓									
Therapist												
Psychologist		✓	✓	✓		✓	✓^#^	✓	✓	✓	✓	✓
Non-psychologist					✓							
Peer educator	✓											
Mode of delivery												
Individual	✓			✓	✓	✓	✓		✓			✓
Group (N per group)		✓ (12)	✓ (4–10)					✓ (10)		✓ (10)	✓ (7)	
Outcomes (with significant improvements)												
Physical		✓		✓	✓				✓		✓	
Psychological	✓		✓	✓	✓	✓*	✓	✓	✓	✓	✓	✓
Affective	✓		✓	✓	✓	✓*			✓	✓		✓
Social	✓			✓		✓*						✓

Cells highlighted in black indicate particular component was not reported.

# Reported as ‘study clinician’.

* No significance test conducted.

##### Education

Nine studies used this technique. Education constituted the introductory section of most interventions including a general overview of the intervention. Information on disease symptomatology and details of treatment options were shared with patients to empower them to orientate themselves to the disease. Moreover, self-management of symptoms, ‘flare-ups’, lifestyle changes and prevention of complications were covered by some psychosocial interventions.

##### Communication skills development

Communications with family, healthcare workers and friends appeared in seven studies. Communication skills were geared towards appropriate ways of expressing one’s emotions to friends/family/health professionals to solicit their support. The recognition of one’s need for help as well as strategies for asking for assistance was emphasized in these interventions. Furthermore, some interventions addressed relational issues such as stigma and discrimination by equipping patients with the skills to communicate their needs to others in a way that will foster respect and dignity. Patients were also informed about their basic human rights to enable them to recognize when they are being discriminated against.

##### Relaxation techniques

To deal with the stress of living with chronic diseases, participants were taught skills-based relaxation techniques. Such techniques included mindfulness, attention diversion, engagement in pleasant activities and biofeedback processes such as paced breathing, deep breathing and progressive muscle relaxation. Relaxation techniques were usually practised through the entire course of the intervention as daily homework activities. Seven studies utilized this active technique (see Table 4).

##### Cognitive restructuring

Seven studies incorporated cognitive restructuring into their intervention. The cognitive restructuring included techniques that entreated participants to re-examine their thoughts on their current disease condition, identify negative thoughts and attempt to change such thoughts. Cognitive restructuring strategies used included automatic thought recording where patients were asked to record and challenge all intrusive negative thoughts such as catastrophizing and rumination. Moreover, forms of positive reappraisal such as benefit finding were used to change patients’ perceptions and to increase positive affect.

##### Homework

Some psychosocial interventions (*n* = 6) assigned participants with tasks to be completed at home in between sessions. Some of these tasks were intended to reinforce the adaptive skills taught in previous sessions. Other tasks required reflection and keeping a daily record of one’s thoughts, emotions and other psychosocial responses. Homework activities either remained constant throughout the intervention or additional tasks were added to existing homework activities after each session.

##### Problem solving

This intervention facet focused on real-life scenarios and how patients could be guided to pursue the best course of action in such situations. Emphasis was placed on dealing with the daily hassles and situational challenges of the disease. Hands-on practical skills for dealing with anticipated and unanticipated psychosocial challenges of the chronic disease were taught. Five studies mentioned this technique.

##### Acceptance and finding meaning

This component highlighted the acceptance of disease-related limitations imposed on patients as well as the instillation of hope and a sense of purpose in the lives of patients. One study illustrated acceptance of disease with a metaphor of struggling in quicksand where resistance tended to worsen one’s situation. Patients were encouraged to eschew denial and fully embrace their diseased state. Patients were enabled to create new future life plans, amend existing plans or devise novel strategies for attaining their life goals and asked to write down these life goals to serve as daily reminders.

##### Goal setting

Patients were advised on how to set small attainable goals. Such goals were intended to incite a sense of accomplishment in patients. Goal setting involved making a list of tasks to be achieved within a timeframe, and tracking progress made in attaining each goal. Specific details on how goals were set were not available. There was no information on whether goals were set by patients alone or through an agreement between patient and therapist.

#### Other intervention facets

##### Timing of intervention

The mean number of intervention sessions for all studies was 7.6. Studies with fewer sessions than the average were labelled ‘brief’ (*n* = 7) whereas those with more sessions were classified as ‘long-term’ as shown in Table 4. The median time between each session was 1 week (range 1–5 weeks) and each session lasted for 60 minutes (range 17.5–120 minutes). The median duration of intervention was 8 weeks (range 3–24 weeks).

##### Mode of delivery

All interventions were delivered either in individual (*n* = 7) or group format (*n* = 5). One study used a mixture of face-to-face contacts and telephone calls to deliver the intervention (Molton et al., 2019). Groups consisted of a mean of nine participants each (range 4–12). One study clustered participants according to their age groups to allow for the sharing of similar and more meaningful experiences (Calandri et al., 2017).

##### Location

Intervention sites were classified as medical (at a health facility) or non-medical (outside health facility). For most studies (*n* = 7), intervention took place at the hospital, clinic or other health facilities. One study reported the use of a non-health facility centre in the form of a castle with a park as the site of intervention (Calandri et al., 2017). Two studies did not report the location of their intervention (Brusadelli et al., 2018; Moskowitz et al., 2017).

##### Therapist, training and fidelity

Three categories of interventionists were identified – psychologists (*n* = 9), non-psychologists (*n* = 2) and peer educators (*n* = 1). In one study, the intervention was delivered by facilitators with knowledge in public health research (Moskowitz et al., 2017). Another study was unclear about the qualification of the interventionists, naming a ‘study clinician’ as the therapist (Molton et al., 2019). In addition, one study used peers, not as the main interventionist, but as language and cultural support staff to assist the psychologist (Mundell et al., 2011).

Only two studies provided details of therapist training. The training focused on giving information on disease-specific issues and equipping therapists with facilitation skills through role-playing prior to intervention. Measures to ensure strict adherence to intervention protocols were reported in only three studies. Fidelity was maintained by qualitatively reviewing audio recordings of treatment sessions and organizing debriefing meetings among facilitators to resolve emerging issues after each session.

##### Questionnaires used for psychosocial outcomes

Thirty-three questionnaires were used by various studies to measure psychosocial outcomes as shown in Table 5. Most of those assessment instruments were used to measure depression, anxiety and affect (*n* = 9). The Centre for Epidemiological Studies Depression Scale (CES-D) was the most widely used instrument (*n* = 3). A majority of the remaining questionnaires were used in only two studies. This included the Beck’s Depression Inventory (BDI), Patient Health Questionnaire (PHQ), Hospital Anxiety and Depression Scales (HADS), State Trait Anxiety Inventory (STAI), Acceptance of Chronic Health Conditions Scale (ACHC) and Short Form Health Survey (SF-12). The remaining instruments were used only once.

**Table 5.** Questionnaires used for measuring psychosocial outcomes.

No.	Questionnaire	References	Outcome measured	Description	Studies in which they were used
1	Center for Epidemiologic Studies Depression Scale (CES-D)	Radloff (1977)	Depression	A 20-item scale designed to assess depressive symptoms (past week) in the general population, rather than in the population of people clinically diagnosed with depression	Brashers et al. (2017), Moskowitz et al. (2017) and Mundell et al. (2011)
2	10-item Center for Epidemiologic Studies Depression Scale (CES-D-10).	Andresen et al. (1994)	Depression	A short version of the 20-item CES-D. The CES-D-10 measures the frequency of depressive symptoms during the past week	Calandri et al. (2017)
3	Beck Depression Inventory-I/II (BDI)	Beck et al. (1996)	Depression	This is a 21-item self-reporting questionnaire for evaluating the severity of depression in normal and psychiatric populations	Kiropoulos et al. (2016) and Spiess et al. (1995)
4	Patient Health Questionnaire (PHQ-9)	Kroenke et al. (2001)	Depressive symptoms	The 9-item PHQ-9 was specifically developed for use in primary care settings for making diagnosis of depression in such settings.	Moitra et al. (2015) and Yang et al. (2018)
5	Hospital Anxiety Depression Scale (HADS)	Zigmond and Snaith (1983)	Anxiety and depression	A 14-item questionnaire developed to assess anxiety and depression in patients with physical health problems	Brusadelli et al. (2018) and Sharpe et al. (2001)
6	State Trait Anxiety Inventory (STAI)	Spielberger et al. (1970)	Anxiety	The 20-item STAI evaluates feelings of tension, nervousness, worry and apprehension ‘in the past 2 weeks, including today’ with higher scores reflecting higher severity	Kiropoulos et al (2016) and Spiess et al. (1995)
7	Generalized Anxiety Disorder – 7 (GAD-7)	Spitzer et al. (2006)	Global anxiety	This questionnaire is a 7-item, self-report anxiety questionnaire designed to screen and assess the severity of generalized anxiety during the previous 2 weeks.	Molton et al. (2019)
8	Positive Affect Negative Affect Schedule (PANAS)	Watson et al. (1988)	Positive affect, negative affect	The schedule constitutes two mood scales, one measuring Positive Affect (PA) (10 items) and the other measuring Negative Affect (NA) (10 items)	Calandri et al (2017)
9	Differential Emotions Scale (DES)	Fredrickson et al. (2003)	Positive and negative affect	Assesses nine positive emotions (amused, awe, content, glad, grateful, hopeful, interested, love and pride) and eight negative emotions (angry, ashamed, contempt, disgust, embarrassed, repentant, sad and scared)	Moskowitz et al. (2017)
10	Life Orientation Test-Revised (LOT-R)	Scheier et al. (1994)	Optimism	The test comprises 10 items (three items framed in a positive way, three items framed in a pessimistic way and four fillers to disguise the purpose of the test). It measures future expectations that are either positive or negative	Calandri et al (2017)
11	Ways of Coping Questionnaire (WCQ)	Folkman and Lazarus (1988)	Coping	The 66-item tool consists of eight scales measuring confrontive coping, distancing, self-controlling, seeking social support, accepting responsibility, escape-avoidance, planful problem-solving and positive reappraisal.	Kiropoulos et al (2016)
12	Brief Coping Orientation to Problems Experienced (Brief COPE)	Carver (1997)	Coping	This tool has 14 subscales that assess acceptance, emotional social support, humour, positive reframing, religion, active coping, instrumental support, planning, behavioural disengagement, denial, self-distraction, self-blaming and substance use and venting.	Mundell et al. (2011)
13	Coping Strategy Questionnaire (CSQ)	Rosenstiel and Keefe (1983)	Coping strategies	It includes seven subscales – two involve maladaptive strategies and the remaining five involve adaptive strategies. A total score of active coping is calculated by subtracting the passive scale scores from the sum of the active scale.	Sharpe et al. (2001)
14	Brief Adjustment Scale (BASE-6)	Cruz et al. (2020)	Problems in psychological adjustment	It assesses emotion distress (depression, anxiety and anger) and related interference (impact on self-esteem, personal relationships and occupational functioning)	Yang et al. (2018)
15	Illness Uncertainty Scale (IUS)	Mishel (1981)	Uncertainty	Evaluates the three aspects of uncertainty experience: ambiguity, complexity and deficiency of information	Brashers et al. (2017)
16	Intolerance of Uncertainty scale	Buhr and Dugas (2002)	Intolerance of uncertainty	This 27 items instrument is related to the idea that uncertainty is unacceptable, leads to frustration and creates an inability to take action.	Molton et al. (2019)
17	Hackett-Cassem Denial Scale	Hackett and Cassem (1974)	Denial	31-item scale used to quantify denial traits and to classify individuals into mild, moderate and major deniers	Spiess et al. (1995)
18	Acceptance of Chronic Health Conditions Scale (ACHC)	Stuifbergen et al. (2008)	Acceptance or psychological flexibility	The 10-item ACHC scale measures acceptance to chronic disease	Kiropoulos et al. (2016) and Molton et al. (2019)
19	Acceptance and Action Questionnaire–II measure (AAQ-II)	Bond et al. (2011)	Acceptance	The AAQ-II assesses general psychological acceptance, emotional willingness and tendency to engage in experiential avoidance	Moitra et al. (2015)
20	33-item Resilience Scale for Adults (RSA)	Friborg et al. (2005)	Resilience	The 33 items in the scale cover six dimensions namely: Perception of self, Planned future, Social competence, Structured style, Family cohesion, Social resources	Kiropoulos et al (2016)
21	Social Support Scale (SSS)	Brashers et al. (2017)	Social support	This 6-item scale assesses the number of supportive others and level of satisfaction participants’ have with their support	Brashers et al. (2017)
22	Perceived Social Support Scale (PSSS)	Blumenthal et al. (1987)	Perceived social support	The 12-item version of the PSSS measures an individual’s perceptions of the social support and emotional closeness with peers, families and other interpersonal relations	Kiropoulos et al (2016)
23	Multidimensional Social Support Inventory	Bauman and Weiss (1995)	Perception of social support	The instrument was originally developed to assess the five domains of social support among minority women with HIV/AIDS in the USA	Mundell et al. (2011)
24	Medical Outcomes Study – Social Support Scale	Sherbourne and Stewart (1991)	Perceived social support	The scale measures the perceived social support for patients living with chronic illness	Yang et al. (2018)
25	10-item form of the HIV Stigma Scale (HSS)	Berger et al. (2001)	Stigma	The revised 10-item version of the HSS measures stigma as a construct in HIV positive patients	Moitra et al. (2015)
26	Rosenberg Self-Esteem Scale (RSE)	Rosenberg (1986)	Self-esteem	The RSE is a 10-item scale designed to evaluate global self-esteem	Mundell et al. (2011)
27	Short form Health Survey (SF-36)	Ware and Sherbourne (1992)	Physical and psychosocial functioning (Quality of life)	A psychometrically validated questionnaire with 36-items divided among eight scales: Physical Functioning, Role-physical Functioning, Bodily Pain, General Health Perceptions, Vitality, Social Functioning, Role-emotional Functioning and Mental Health.	Visschedijk et al. (2004)
28	Short Form Health Survey (SF-12)	Jenkinson et al. (1997)	Physical and Psychosocial functioning (Quality of life)	This survey is the validated and short version of the SF-36 used for assessing health status. It is composed of 12 items that provide measures of Physical Health (PCS) and Mental Health (MCS),	Brusadelli et al. (2018) and Calandri et al (2017)
29	Multiple Sclerosis Quality of Life (MSQOL-54)	Vickrey et al. (1995)	MS-related quality of life	The 54-item questionnaire measures quality of life using 12 subscales: physical function, role limitations-physical, role limitations-emotional, pain, emotional well-being, energy, health perceptions, social function, cognitive function, health distress, overall quality of life and sexual function.	Kiropoulos et al. (2016)
30	Patient Self-Advocacy Scale (PSAS)	Brashers et al. (1999)	Self-advocacy	The scale assesses three dimensions of patient-provider interactions: (1) education, (2) assertiveness (3) nonadherence.	Brashers et al. (2017)
31	15-item Impact of Event Scale	Horowitz et al. (1979)	Intrusive and Avoidant thought	The scale assesses subjective distress resulting from exposure to stressful life situations.	Moskowitz et al. (2017)
32	Ahrens scale	Ahrens and Elsner (1981)	Attributional belief	The 30-item scale measures attributional belief, that is, internal attribution and external attribution.	Spiess et al. (1995)
33	Junk and Junk, questionnaire	Junk and Junk (1981)	Life events	The scale assesses 70 stressful events that had occurred in the past 6, 12 or more months	Spiess et al. (1995)

##### Effects of interventions on outcomes

Outcome measures used in included studies are as shown in Table 6. Intervention outcomes were classified as physical, psychological, affective and social outcomes.

**Table 6.** Physical, psychological, affective and social outcomes.

Study	Physical outcome		Affective outcome		Psychological outcome		Social outcome	
Sharpe et al. (2001)	+	Pain, C-reactive protein.	++	HADS-D	0	HAD-A.		/
	0	ESR, HAQ.			+	CSQ		
	++	RAI						
Kiropoulos et al. (2016)	++	MFIS, PES, MSQOL physical health, PSQI.	++	BDI-II	+	STAI.	++	PSSS
					++	MSQOL mental health, RSA.		
Spiess et al. (1995)	0	HbA1c.	+	BDI	+	Quality of Coping.		/
					++	Denial.		
					0	Attributional belief.		
Visschedijk et al. (2004)	+	SF-36 (vitality subscale).		/	0	SF-36 (Mental health subscale).		/
	0	Disability and Impact profile (Mobility and self-care).			+	Disability and Impact profile (Psychological status).		
Brusadelli et al. (2018)	+	HbA1c.	/	HADS-D.	/	HADS-A, SF-12.		/
Calandri et al. (2017)	0	SF-12 (physical health).	+	PANAS	++	SF-12 (mental health), LOT-R.		/
			0	CES-D-10.				
Moitra et al. (2015) *		/	/	PHQ-9.	/	AAQ, HCSD.		/ HSS
Molton et al. (2019)		/		/	++	Intolerance to uncertainty, ACHC-MS.		/
					+	GAD-7		
Moskowitz et al. (2017)	0	CD4 and viral load.	+	DES.	++	Impact of Event Scale		/
	++	Symptom severity.	0	CES-D.				
Yang et al. (2018)		/	++	PHQ-9.	++	BASE-6, distress and coping.	++	Social support
Brashers et al. (2017)		/	+	CES-D.	+	Illness uncertainty scale.	+	Social support
					0	Patient Self-Advocacy Scale.		
Mundell et al. (2011)		/	0	CES-D	+	Brief COPE.	0	Multidimensional Social Support Inventory
					0	Rosenberg Self-Esteem Scale.		

* No test of significance; **0:** No statistically significant improvement; **+:** Conditionally significant (only significant at some follow-up point or significant improvement for both control/treatment groups or only significant in some dimensions of the scale but not others); **++:** Statistically significant; **/:** Not reported;

Meaning of the abbreviations used here can be found in Table 5.

Physical outcomes comprised of physical health subscales of the generic SF-12 and Quality of life scales (QoL). In addition, studies reported disease-specific measures such as pain, C-reactive protein, Health assessment questionnaire (HAQ) scores, Joint dysfunction and Erythrocyte Sedimentation Rate (ESR) in RA; blood glucose levels (HbA1c) in T1D and T2D; disability scores in MS and Viral load and CD4 count in HIV. Physical outcomes were reported in seven studies, five of which demonstrated a statistically significant improvement in at least one physical outcome. Three out of the five CBTs, the PAI and one study with unspecified theory produced improvement in physical outcomes.

Reported affective outcomes included depression, positive affect, negative affect and affective well-being. Eight studies reported at least one affective outcome measure. The majority of studies (*n* = 7) reported a statistically significant improvement in at least one outcome. Four out of the five studies utilizing CBTs reported a decline in negative affect and/or depression and improvement in positive affect. Each of the PAI and UMI studies also produced improvements in affective outcome.

Social outcomes were scarcely reported with four studies reporting this outcome. Social outcomes consisted of perceived social supports, satisfaction with social support, quality of life subscale (social health). All but one (Mundell et al., 2011) of the four studies reported significant improvements in these outcomes. The UMI study and only two out of the five CBT studies had significant improvements in social outcomes.

Psychological outcomes included acceptance, coping, uncertainty, self-esteem, anxiety, denial, attributional beliefs, optimism, psychological adjustment, psychological distress, quality of life subscale, the impact of life event and resilience. Psychological outcomes were reported in all but one study (Brusadelli et al., 2018). The Brusadelli et al paper did not report the results of their affective and psychological outcome measures. The Moitra et al, study reported psychosocial outcomes but failed to conduct appropriate significance tests on these outcomes. Statistically significant improvements were seen in all studies that reported psychological outcomes. The studies that recorded such improvements included all studies using CBT, PAI, UMI, UI and SSI. Even though no significance test was done, the Moitra et al. study showed an improvement in all psychosocial outcomes post-intervention.

## Discussion

The review found CBT, ACT, PAI, SSI and UMI as the existing interventions for adults with newly diagnosed chronic diseases. Most interventions were grounded in theories such as Beck’s cognitive theory for CBT, Hayes’ relational frame theory for the ACT, Positive emotions theory for PAI, uncertainty management theory for UMI and systems theory for SSI. Theories provide insight into the underlying mechanisms of change for the psychosocial intervention (Craig et al., 2008).

Second wave cognitive behavioural therapy (CBT) and its variants (CBT-like or third-wave CBTs) were the predominant intervention types. This finding was not unexpected given CBT’s status as the gold standard of psychotherapy (David et al., 2018) and the most widely studied psychological intervention (Hofmann et al., 2012). CBT has been successfully applied to patients with long-standing T1D (McGrady and Hood, 2013), T2D (Safren et al., 2014), MS (Hind et al., 2014), HIV (Tobin et al., 2017), RA (Sharpe, 2016) and Inflammatory bowel disease (IBD) (Mikocka-Walus et al., 2015).

Positive affect interventions (PAI) are generally considered members of the CBT family (Forman and Herbert, 2009; Prasko et al., 2016). While traditional CBT focuses on altering negative thoughts, PAI rather seeks to heighten individuals’ daily experience of positive emotions and reverse lingering negative emotions by enabling patients to focus on their strengths and to see life beyond their current condition (Prasko et al., 2016) as suggested by the broaden-and-build theory of positive emotions (Fredrickson, 2004). Much emphasis was placed on optimism and other positive psychological constructs in PAI as studies have shown that these positive emotions are independently associated with improvement in health outcomes (Chida and Steptoe, 2008).

The relational frame theory, which was foundational to ACT, focused on enhancing psychological flexibility in order to promote acceptance to chronic diseases (Hayes et al., 2001; Kuba and Weißflog, 2017). In similar studies, ACT has shown effectiveness in long-standing MS (Gillanders and Gillanders, 2014), type 2 diabetes (Gregg et al., 2007) and other chronic diseases (Graham et al., 2016; Nordin and Rorsman, 2012) for relieving disease-related distress, improving self-management practices and psychological flexibility respectively.

The uncertainty theory asserts that uncertainty is a neutral experience (neither positive nor negative), that is, not always linked to anxiety and that although it may be perceived as threatening for some individuals, uncertainty may also incite hope and optimism in others. Therefore, some individuals may seek to reduce their level of uncertainty while others will attempt to maintain it. The goal of the uncertainty management intervention was thus to equip individuals with adaptable communication skills for them to apply as needed to manage their uncertainty.

Most interventions produced the results predicted by their underlying theories. For CBT, significant improvements were seen in depression as predicted by the cognitive theory, whereas for PAI optimism and positive affect score increased as predicted by the positive emotions theory. The level of uncertainty and acceptance of chronic disease also improved in the ACT study in concordance with relational frame theory. Improvements in these outcomes facilitated psychosocial adaptation (Bury, 1982; De Ridder et al., 2008; Due-Christensen et al., 2018). However, most CBT studies were of low quality; hence interpretation of the improvements in the outcomes should be done cautiously.

The review found education, communication skills development, relaxation techniques, cognitive restructuring, homework, problem-solving, acceptance and finding meaning and goal setting as the active techniques employed by psychosocial interventions for adults with newly diagnosed chronic disease. Majority of the psychosocial interventions identified in this review utilized techniques that reflected their theoretical underpinnings. For instance, cognitive restructuring and relaxation techniques featured prominently in studies that used traditional CBT interventions whereas acceptance and finding meaning were mostly used in third-wave CBT interventions such as ACT.

Each active technique addressed a specific facet of the adaptation process. For instance, cognitive restructuring was used to change negative thoughts or illness perception which is known to influence psychosocial adaptation to chronic disease (Kocaman et al., 2007; Lazarus and Folkman, 1984; Leventhal et al., 2016). Similarly, education and communication skills were used to reduce uncertainty to the disease, consistent with the psychosocial adaptation literature (Carleton, 2016; Folkman, 2010; Visschedijk et al., 2004). Acceptance and finding meaning also made it possible for patients to deal with grief due to loss of health by giving patients hope and assurance that all is not lost. Other techniques such as goal setting and problem-solving have also been recognized as instrumental in coping with chronic diseases (Hall and Foster, 1977; Scobbie et al., 2009).

Homework activities were geared towards reinforcing learned skills and enabling patients to normalize such skills to their daily lives. Homework thus addressed the dynamic and ongoing nature of psychosocial adaptation (Helgeson and Zajdel, 2017; Stanton et al., 2007). Consistent with other studies, this technique was mostly used in CBT to improve psychosocial outcomes (Cronin et al., 2015; Hayasaka et al., 2015).

The active techniques appeared in varying doses across the various psychosocial interventions. Due to the heterogeneity of outcome measures, meta-regression analysis could not be performed to determine the association between the number of active techniques (dosage) and type of techniques used by the studies and the effect that had on specific outcome measures (effect size). Notwithstanding, one could argue that psychosocial interventions having more active techniques (across various theoretical frameworks) are more likely to achieve positive psychosocial outcomes as the varied techniques could help address all facets of psychosocial adaptation. It is worth noting that there is an ongoing debate about whether this eclectic approach is superior to other approaches that advocates for strict adherence to a particular theoretical framework (Zarbo et al., 2016). Clinical trials comparing the effectiveness of these two approaches may help bring finality to this debate. It is also important to note that the heterogeneous outcomes reported by the included studies did not allow for conclusions to be drawn about the intervention techniques that were most effective.

Although we did not focus on non-specific techniques in psychosocial interventions, we acknowledge that such techniques are useful in psychotherapy. A typical non-specific technique identified in the review was group dynamics which generally include cohesiveness, modelling and group bond (Chatoor and Krupnick, 2001). Group therapies are effective if participants have common characteristics that allow them to share their experiences. However, as found in the current review, some participants experienced isolation or perceived rejection from the group resulting in attrition – they were worried about being the most disabled or least disabled patients in group sessions (Visschedijk et al., 2004). The non-random dropout of participants might have caused an overestimation of the intervention effect in the study.

The interventionists in most studies received no training. It is likely that the researchers being the developers and implementers of the interventions coupled with the fact that most therapists were professional psychologists, meant that no special training was required. Nonetheless, developing training for interventionists would have enhanced the ability for others to fully replicate the various studies and could have improved the quality of the studies. Although fidelity of intervention protocol was explicitly reported by only three studies, other studies hinted on fidelity by mentioning the use of treatment manuals (Kiropoulos et al., 2016; Sharpe et al., 2001). Treatment manuals spell out the requisite elements of an intervention and are usually used as the basis for assessing fidelity (Horner et al., 2006). Similar reviews have found the lack of fidelity adherence in most psychosocial interventions (McArthur et al., 2012). The lack of treatment fidelity may cause potentially effective interventions to appear ineffective and lead to faulty conclusions (McArthur et al., 2012). On the other hand, an intervention could appear effective because of the skills of the psychologists delivering the intervention – but not effective if delivered by someone else using the same treatment protocol.

This review excluded several studies that used educational and/or self-management interventions to improve psychosocial outcomes in T1D and T2D because such interventions failed to describe clearly their psychological components. At best, some of these interventions hinted at the use of psychological techniques but did not provide enough details to allow for their inclusion in the current review. Future studies should be explicit about all intervention components as recommended by Hodges et al. (2011).

The review did not find psychosocial interventions for newly diagnosed IBD patients. Patients with IBD have several psychosocial concerns ranging from impaired body image to stigmatization that warrant special attention (Sajadinejad et al., 2012). Although interventions exist to support IBD patients, such interventions target patients with longer disease duration (>3 years) (Timmer et al., 2008). The early years of diagnosis are considered formative (Due-Christensen et al., 2018) and interventions targeting this period are likely to produce desirable results. The development of interventions for adults newly diagnosed with IBD should, therefore, be of interest for future research.

## Strengths and limitations

One strength of this review is its focus on six chronic diseases that ensured evidence synthesis across several distinct disease domains spanning from infectious diseases (such as HIV) to autoimmune diseases (such as RA) to produce a more comprehensive picture than we would have produced if we had focused on one chronic disease. Another strength of this review was the involvement of three reviewers which ensured transparency and objectivity in the selection and synthesis of evidence.

The study had some limitations. The current review excluded grey literature and papers that were not written in the English language. This could have resulted in the loss of valuable evidence from non-English speaking countries as well as evidence from unpublished studies. Nonetheless, the inclusion of five electronic databases in the search strategy could be regarded as a strength of this review as it increased the search coverage and the likelihood of capturing the most relevant papers.

The studies included in this review were of low to moderate quality with some having low internal and external validity. The effect of the interventions on psychosocial and physical outcomes should, therefore, be interpreted with caution.

## Recommendations

Researchers should take measures to strengthen the quality of future studies involving psychosocial interventions for adults with newly diagnosed chronic disease. Such measures may include the use of larger samples, a control group and a measure of effect size to allow for firm conclusions to be drawn regarding the effects of the intervention. Future studies should also adhere to known frameworks for developing complex interventions such as the well-accepted Medical and Research Council’s framework (Craig et al., 2008). This will help prevent some of the issues seen in this review such as the lack of fidelity adherence in studies. We also recommend that researchers adhere to the COMPASS checklist when reporting details of their intervention methods and delivery to allow for replicability of their studies.

It is worth noting that some patients may feel too distressed about their new diagnosis and may not be immediately receptive of any intervention. Future research should focus on identifying the time after diagnosis, that is, optimal to initiate psychosocial interventions for patients with chronic disease. In addition, the current review found several heterogeneous outcome measures that made meta-analysis and other further analysis impossible. Future researchers should, therefore, consider developing core outcome measures (COM) for interventions that are intended to facilitate psychosocial adaptation in adults with newly diagnosed chronic disease. The COM would stipulate the minimum set of outcomes required to evaluate psychosocial interventions and would make evidence synthesis across multiple studies seamless (Williamson et al., 2012).

The few studies (*n* = 12) we found for the current review suggests a dearth of research in the area of psychological interventions for adults with newly diagnosed chronic disease. This is inconsistent with the widely accepted need to provide support for such patients to facilitate adjustment to their diagnosis (De Ridder et al., 2008; Due-Christensen et al., 2019; Stanton et al., 2007). More funding is thus required to stimulate more research in this area of supporting adaptation in the early stages of chronic disease diagnosis. More so, none of the studies we found was from poorly resourced countries where psychosocial issues are often not addressed. It may be useful to develop and evaluate future interventions in such poorly resourced areas. Perhaps, a strategy to increase psychosocial interventions in such settings will be to use peer educators as studies have proven such therapists to be cheaper but comparatively effective alternatives (Andreae et al., 2020).

This synthesis of active ingredients of psychosocial interventions across six chronic diseases is the first of its kind. However, some studies have examined related areas (such as behavioural change interventions) to identify the active techniques of such interventions (Cradock et al., 2017). Indeed, a set of taxonomy exists for behavioural change techniques from which researchers can draw to develop their interventions (Michie et al., 2013). The synthesized active ingredients in the current review could perhaps be a stepping stone for future researchers to develop a similar standardized taxonomy that can inform the development of novel psychosocial interventions for adults with newly diagnosed chronic disease. This taxonomy would allow for more consistent and standardized definition and application of psychosocial interventions.

## Conclusions

Several active techniques were employed in the various psychosocial interventions to aid adjustment to newly diagnosed chronic disease. The evidence synthesized in this review will be useful in future intervention development as well as in the standardization of psychosocial interventions for adults with newly diagnosed chronic disease.

## Supplemental Material

sj-pdf-1-hpq-10.1177_1359105321995916 – Supplemental material for Psychosocial interventions for adults with newly diagnosed chronic disease: A systematic review Click here for additional data file. Supplemental material, sj-pdf-1-hpq-10.1177_1359105321995916 for Psychosocial interventions for adults with newly diagnosed chronic disease: A systematic review by Samuel Akyirem, Angus Forbes, Julie Lindberg Wad and Mette Due-Christensen in Journal of Health Psychology

sj-pdf-2-hpq-10.1177_1359105321995916 – Supplemental material for Psychosocial interventions for adults with newly diagnosed chronic disease: A systematic review Click here for additional data file. Supplemental material, sj-pdf-2-hpq-10.1177_1359105321995916 for Psychosocial interventions for adults with newly diagnosed chronic disease: A systematic review by Samuel Akyirem, Angus Forbes, Julie Lindberg Wad and Mette Due-Christensen in Journal of Health Psychology
