# Supplementary material for: Psychosocial interventions for adults with newly diagnosed chronic disease: A systematic review
Source: J Health Psychol. 2021 Feb 14;27(7):1753–82. doi: 10.1177/1359105321995916 (PMC9092922; doi:10.1177/1359105321995916)
Supplement: sj-pdf-2-hpq-10.1177_1359105321995916 – Supplemental material for Psychosocial interventions for adults with newly diagnosed chronic disease: A systematic review [file sj-pdf-2-hpq-10.1177_1359105321995916.pdf]

Full search strategy on Medline (20th May 2020)

#	Searches	Results
1	exp HIV/	98222
2	"HIV".mp.	356525
3	exp HIV Infections/	280875
4	"HIV infection".mp.	66731
5	"HIV/AIDS".mp.	30987
6	"Retroviral infection".mp.	1219
7	"PLWH".mp.	1575
8	"PLWHA".mp.	926
9	exp Arthritis, Rheumatoid/	112384
10	"Rheumatoid arthritis".mp.	105311
11	"Rheumatism".mp.	14677
12	"arthritis".mp.	220162
13	"Arthralgia".mp.	13814
14	exp Diabetes Mellitus/	422060
15	"diabetes mellitus".mp.	431578
16	diabetes.mp.	615473
17	"type 1 diabetes".mp.	40054
18	"type 2 diabetes".mp.	125871
19	"T1DM".mp.	4585
20	"T2DM".mp.	20416
21	exp Multiple Sclerosis/	58217
22	"multiple sclerosis".mp.	82579
23	exp Crohn Disease/	38666
24	exp Inflammatory Bowel Diseases/	80234
25	exp Colitis, Ulcerative/	34318
26	Inflammatory bowel disease*.mp.	52473
27	crohn* disease*.mp.	55162
28	ulcerative colitis.mp.	39374
29	IBD.mp.	24983
30	1 or 2 or 3 or 4 or 5 or 6 or 7 or 8 or 9 or 10 or 11 or 12 or 13 or 14 or 15 or 17 or 18 or 19 or 20 or 21 or 22 or 23 or 24 or 25 or 26 or 27 or 28 or 29	1351723
31	"new diagnosis".mp.	2901
32	"newly diagnosed".mp.	48143
33	"recently diagnosed".mp.	3922
34	"recent diagnosis".mp.	796
35	"new onset".mp.	16451
36	"first time diagnosis".mp.	304
37	"time of diagnosis".mp.	29174
38	"after diagnosis".mp.	20127
39	"on diagnosis".mp.	6364
40	31 or 32 or 33 or 34 or 35 or 36 or 37 or 38 or 39	121832
41	exp Cognitive Behavioral Therapy/	28111
42	"cognitive behavio?ral therap*".mp.	30771
43	"acceptance and commitment therapy".mp.	994
44	"psychoeducation*".mp.	4927
45	psychosocial.mp.	97151

46	"patient education".mp.	100043
47	exp Psychotherapy/	194338
48	"psychotherap*".mp.	90132
49	exp Social Support/	70440
50	"social support".mp.	88585
51	"psychological support".mp.	3678
52	"acceptance".mp.	111227
53	"adaptation".mp.	310804
54	resilience.mp.	25591
55	41 or 42 or 43 or 44 or 45 or 46 or 47 or 48 or 49 or 50 or 51 or 52 or 54	588047
56	30 and 40 and 55	1049
57	limit 56 to (abstracts and english language and humans)	911
58	57 not child.mp.	749
59	58 and "Journal Article" [Publication Type]	748

Full search strategy on PyschInfo (May Week 4 2020)

#	Searches	Results
1	exp HIV/ or HIV.mp.	56543
2	HIV infection.mp.	10531
3	exp AIDS/ or "HIV/AIDS".mp.	22400
4	"Retroviral infection".mp.	26
5	"PLWH".mp.	686
6	"PLWHA".mp.	477
7	Rheumatoid arthritis.mp. or exp Rheumatoid Arthritis/	2849
8	"Rheumatism".mp.	251
9	exp Arthritis/ or "arthritis".mp.	6838
10	"Arthralgia".mp.	321
11	diabetes mellitus.mp. or exp Diabetes Mellitus/	14238
12	"type 1 diabetes".mp.	2027
13	"type 2 diabetes".mp. or exp Type 2 Diabetes/	8183
14	"T1DM".mp.	247
15	"T2DM".mp.	972
16	Multiple sclerosis.mp. or exp Multiple Sclerosis/	16256
17	exp Ulcerative Colitis/	295
18	crohn* disease*.mp.	677
19	ulcerative colitis.mp.	672
20	IBD.mp.	542
21	Inflammatory bowel disease*.mp.	978
22	1 or 2 or 3 or 4 or 5 or 6 or 7 or 8 or 9 or 10 or 11 or 12 or 13 or 14 or 15 or 16 or 17 or 18 or 19 or 20 or 21	98087
23	"new diagnosis".mp.	475
24	"newly diagnosed".mp.	3530
25	"recent diagnosis".mp.	159
26	"recently diagnosed".mp.	859
27	"new onset".mp.	1751
28	"first time diagnosis".mp.	47
29	"time of diagnosis".mp.	2130
30	"after diagnosis".mp.	1770

31	"on diagnosis".mp.	1247
32	23 or 24 or 25 or 26 or 27 or 28 or 29 or 30 or 31	11105
33	exp Cognitive Therapy/ or exp Cognitive Behavior Therapy/ or Cognitive behavio?ral therapy.mp.	38876
34	"acceptance and commitment therapy".mp. or exp "Acceptance and Commitment Therapy"/	2420
35	exp Psychoeducation/ or "psychoeducation*".mp.	10741
36	psychosocial.mp. or exp Psychosocial Rehabilitation/	121246
37	"patient education".mp. or exp Client Education/	6082
38	psychotherapy.mp. or exp Psychotherapy/	243657
39	social support.mp. or exp Social Support/	80052
40	psychological support.mp.	1803
41	acceptance.mp.	55256
42	adaptation.mp.	67801
43	resilience.mp.	27839
44	33 or 34 or 35 or 36 or 37 or 38 or 39 or 40 or 41 or 42 or 43	584043
45	22 and 32 and 44	364
46	22 and 32 and 44	364
47	limit 46 to (human and english language and abstracts)	341
48	47 and "Journal" [Publication Type]	286
49	48 not child.mp.	240

Full Search strategy on Embase (2020 Week 21)

#	Searches	Results
1	HIV.mp. or exp Human immunodeficiency virus/	429413
2	HIV infection.mp.	85898
3	exp Human immunodeficiency virus infection/	374174
4	exp acquired immune deficiency syndrome/ or "HIV/AIDS".mp.	160908
5	"Retroviral infection".mp. or exp retrovirus infection/	384360
6	"PLWH".mp.	2158
7	"PLWHA".mp.	1173
8	Rheumatoid arthritis.mp. or exp rheumatoid arthritis/	218554
9	"Rheumatism".mp.	10397
10	"arthritis".mp.	332513
11	"Arthralgia".mp.	63216
12	diabetes mellitus.mp. or exp diabetes mellitus/	983463
13	"type 1 diabetes".mp. or exp insulin dependent diabetes mellitus/	124918
14	"type 2 diabetes".mp. or exp non insulin dependent diabetes mellitus/	290242
15	"T1DM".mp.	9227
16	"T2DM".mp.	36656
17	"multiple sclerosis".mp. or exp multiple sclerosis/	139284
18	exp Ulcerative Colitis/	71526
19	exp Inflammatory Bowel Disease/	147351
20	exp Crohn Disease/	89836
21	ulcerative colitis.mp.	79284
22	crohn* disease*.mp.	98891
23	Inflammatory bowel disease*.mp.	88279

24	IBD.mp.	52399
25	1 or 2 or 3 or 4 or 5 or 6 or 7 or 8 or 9 or 10 or 11 or 12 or 13 or 14 or 15 or 16 or 17 or 18 or 19 or 20 or 21 or 22 or 23 or 24	2178402
26	"new diagnosis".mp.	6209
27	"newly diagnosed".mp.	88242
28	"recent diagnosis".mp.	1676
29	"recently diagnosed".mp.	6796
30	"new onset".mp.	30493
31	"first time diagnosis".mp.	480
32	"time of diagnosis".mp.	56277
33	"after diagnosis".mp.	33382
34	"on diagnosis".mp.	9533
35	26 or 27 or 28 or 29 or 30 or 31 or 32 or 33 or 34	219735
36	exp behavior therapy/ or exp cognitive behavioral therapy/ or exp cognitive therapy/	88195
37	"cognitive behavio?ral therapy".mp.	25061
38	"acceptance and commitment therapy".mp. or exp "acceptance and commitment therapy"/	1769
39	exp psychoeducation/ or "psychoeducation*".mp.	11682
40	psychosocial care/ or mental health care/ or social care/	54740
41	psychosocial.mp.	147425
42	"patient education".mp. or exp patient education/	119859
43	exp psychotherapy/ or psychotherapy.mp.	253492
44	social support.mp. or exp social support/	100874
45	psychological support.mp.	6499
46	acceptance.mp.	93241
47	commitment.mp.	50656
48	exp psychological resilience/ or resilience.mp.	29799
49	36 or 37 or 38 or 39 or 40 or 41 or 42 or 43 or 44 or 45 or 46 or 47 or 48	765098
50	25 and 35 and 49	1526
51	limit 50 to (abstracts and human and english language)	1382
52	51 and "Article" [Publication Type]	790
53	52 not child.mp.	644

Search Strategy on Pubmed (20th May 2020)

#	**Searches**	**Results**
1	((((((((((((((HIV) OR HIV infection) OR "Retroviral infection") OR AIDS) OR HIV/AIDS) OR Acquired immune deficiency syndrome) OR "Human Immuno deficiency virus") OR HIV-1) OR HIV-2) OR PLWH) or PLWHA)) OR ((((rheumatoid arthritis) OR arthritis) OR rheumatism) OR arthralgia)) OR (((((diabetes mellitus) OR type 1 diabetes) OR type 2 diabetes) AND T1DM) OR T2DM)) OR multiple sclerosis) OR (IBD OR Inflammatory bowel disease* OR Crohn disease* OR Ulcerative colitis)	1091890
2	((((((((new diagnosis[Text Word]) OR newly diagnosed[Text Word]) OR recent diagnosis[Text Word]) OR recently diagnosed[Text Word]) OR first time diagnosis[Text Word]) OR new onset[Text Word]) OR time of diagnosis[Text Word]) OR after diagnosis[Text Word]) OR on diagnosis[Text Word]	108242
3	((((((((((cognitive behavioural therapy) OR cognitive behavioral therapy) OR (acceptance and commitment therapy)) OR psychoeducation) AND psychosocial) OR patient education) OR psychotherapy) OR social support) OR psychological support) OR acceptance) OR adaptation) OR resilience	1832210
4	#1 AND #2 AND #3	1182
